# Factors Associated With Depressive Symptom Severity in Older Adults With Type 2 Diabetes Mellitus: A Cross‐Sectional Study

**DOI:** 10.1155/nrp/6665900

**Published:** 2026-10-22

**Authors:** Li-Yun Lee, Chao-Lin Liao

**Affiliations:** ^1^ School of Nursing College of Nursing Chang Gung University of Science and Technology Chiayi Taiwan cgust.edu.tw; ^2^ Department of Nursing Nantou Hospital Ministry of Health and Welfare Nantou Taiwan mohw.gov.tw

**Keywords:** aged, comorbidity, depression, diabetes mellitus, psychological distress, sarcopenia, Type 2

## Abstract

**Background:**

Depressive symptoms are common among older adults with Type 2 diabetes mellitus (T2DM) and may be associated with both physical and psychological factors. However, the combined associations of comorbidity burden, sarcopenia risk, handgrip strength and diabetes distress with depressive symptom severity remain insufficiently examined.

**Objective:**

To identify factors associated with depressive symptom scores among older adults with T2DM.

**Methods:**

A cross‐sectional study using convenience sampling was conducted in a diabetes outpatient clinic in central Taiwan. A total of 170 older adults with T2DM were recruited. Measures included the Charlson comorbidity index, the Sarcopenia Functional Questionnaire, handgrip strength testing, the Diabetes Distress Scale and the Geriatric Depression Scale. Stepwise multiple linear regression was used to identify factors associated with depressive symptom scores.

**Results:**

Depressive symptoms were present in 15.9% of participants. Stepwise multiple linear regression identified diabetes distress, high sarcopenia risk, low handgrip strength and comorbidity burden as significant factors associated with depressive symptom scores. The final model explained 69% of the variance in depressive symptom scores. Diabetes distress showed the strongest association (*β* = 0.71, *p* < 0.001).

**Conclusion/Implications for Practice:**

Diabetes distress, high sarcopenia risk, low handgrip strength and comorbidity burden were associated with depressive symptom severity among older adults with T2DM, with diabetes distress showing the strongest association. Routine assessment of diabetes distress, sarcopenia risk, muscle strength and comorbidity burden may help to identify individuals with more severe depressive symptoms and inform timely supportive care.

## 1. Introduction

The global prevalence of diabetes continues to increase. According to the International Diabetes Federation, approximately 589 million adults aged 20–79 years were living with diabetes worldwide in 2024, and this number is projected to rise to 853 million by 2050 [1]. Older adults represent an important population in diabetes care because the combined effects of ageing, long‐term disease progression and diabetes‐related complications may increase vulnerability to disability, functional decline and the need for long‐term care [2]. Therefore, the healthcare needs of older adults with Type 2 diabetes mellitus (T2DM) have become an important public health and clinical concern worldwide.

In addition to physical illness, individuals with T2DM frequently experience psychological distress. Diabetes distress refers to the emotional burden arising from long‐term disease management, self‐care demands and treatment requirements. It may manifest as frustration, anger, anxiety, sadness and feelings of helplessness [3]. Previous studies have shown that approximately one‐third of people with diabetes experience varying degrees of diabetes distress [4, 5]. Ongoing dietary control, medication adherence, physical activity, blood glucose monitoring, communication with healthcare professionals and insufficient support from family and friends may contribute to emotional burden, treatment‐related distress and interpersonal distress among people with diabetes [3, 5]. Depressive symptoms are also common among individuals with T2DM, with previous studies reporting prevalence estimates of approximately 13%–33% among older adults with T2DM [6–8]. Depressive symptoms have been associated with poorer treatment adherence, less optimal self‐management behaviours, poorer glycaemic control, diabetes‐related complications, cardiovascular disease and lower quality of life. Diabetes distress has also been closely associated with depressive symptoms, with higher levels of diabetes distress corresponding to greater depressive symptom severity [5, 9, 10]. Therefore, early identification of depressive symptoms and their associated factors among older adults with T2DM is clinically important for promoting psychological well‐being and improving diabetes care.

In addition to demographic and clinical factors, physical function decline is an important concern among older adults with T2DM. Sarcopenia and sarcopenia‐related functional decline have increasingly been recognised as common health problems in this population [8, 11, 12]. Sarcopenia is characterised by age‐related declines in skeletal muscle mass, muscle strength and physical performance, and several diabetes‐related mechanisms may contribute to its development [11, 13]. Previous studies have reported that sarcopenia is common among individuals with diabetes, with prevalence estimates of approximately 22%–23% [12, 14]. Studies using screening tools such as the Sarcopenia Functional Questionnaire (SARC‐F) have also shown that a substantial proportion of patients with diabetes are at risk of sarcopenia, with estimated prevalence ranging from 18.3% to 29% [15, 16]. Importantly, sarcopenia risk and muscle strength may also be relevant to depressive symptoms among older adults with T2DM. Ida et al. [15] found that depressive symptom scores were significantly associated with sarcopenia risk among older adults with diabetes. Handgrip strength, a commonly used indicator of muscle strength, is included in sarcopenia assessment algorithms and provides a clinically feasible measure of physical function in older adults [13]. Existing evidence suggests that sarcopenia, sarcopenia risk and sarcopenia‐related physical function decline are associated with greater depressive symptom severity among older adults and individuals with diabetes [17–19].

Taken together, older adults with T2DM face multiple challenges related to disease management, psychological distress, comorbidity burden and physical function decline. Although previous studies have examined diabetes distress, sarcopenia, comorbidities and depressive symptoms separately, relatively few have simultaneously investigated diabetes distress, sarcopenia risk, handgrip strength and comorbidity burden in relation to depressive symptom severity among older adults with T2DM. Therefore, this study investigated factors associated with depressive symptom severity among older adults with T2DM, with particular emphasis on diabetes distress, sarcopenia risk, handgrip strength and comorbidity burden. These findings may provide an empirical basis for clinical assessment, early screening and supportive care planning for this population.

## 2. Materials and Methods

### 2.1. Study Design and Setting

This study had a cross‐sectional correlational design. Participants were recruited from the diabetes outpatient clinic of a teaching hospital in central Taiwan. The hospital provides regular outpatient care and follow‐up services for patients with T2DM. The target population comprised older adults with T2DM who were receiving care at this outpatient clinic. This study was reported in accordance with the Strengthening the Reporting of Observational Studies in Epidemiology (STROBE) checklist for cross‐sectional studies.

### 2.2. Participants and Sample Size

The inclusion criteria were as follows: (a) a confirmed diagnosis of T2DM; (b) age 65 years or older; (c) being conscious and alert; and (d) the ability to communicate effectively in Mandarin Chinese or Taiwanese. Participants were excluded if they had been diagnosed with severe cognitive impairment, major depressive disorder, or dementia.

The sample size was estimated using G∗Power 3.1 for linear multiple regression. Because no directly comparable study had simultaneously examined diabetes distress, sarcopenia risk, handgrip strength and comorbidity burden among older adults with T2DM, the parameter setting was informed by Yang et al. [5], who examined the associations among diabetes complications, diabetes distress and depressive symptoms in patients with T2DM using regression‐based analyses. A medium effect size was adopted according to Cohen’s convention (*f* ^2^ = 0.15). With an alpha level of 0.05, a statistical power of 0.80, and nine candidate independent variables, the minimum required sample size was estimated at 114 participants. Given the possibility of incomplete responses, 180 participants were recruited, and 170 were included in the final analysis.

### 2.3. Data Collection Procedures

Data were collected between June 15, 2022, and December 31, 2022. After permission was obtained from the hospital, the researchers recruited eligible participants from the diabetes outpatient clinic. The study purpose, procedures, voluntary nature of participation and confidentiality measures were explained to each participant before enrolment. Written informed consent was obtained before data collection.

Participants completed a self‐administered questionnaire in a private setting and underwent three handgrip strength measurements. In addition, HbA1c data were extracted from participants’ medical records. For each participant, the most recent HbA1c value available at the time of enrolment was recorded as an indicator of glycaemic control. The entire data collection process took approximately 20 min.

### 2.4. Ethical Considerations

This study was reviewed and approved by the hospital’s Human Experimentation Ethics Committee (IRB No. 111028). Participation was voluntary and participants were informed that they could withdraw from the study at any time without affecting their right to receive medical care or treatment.

To protect confidentiality, each participant was assigned a unique study code, and no personal identifiers were entered into the analytical dataset. Questionnaire data and extracted medical record data were linked only by the study code. Paper‐based data were stored in a locked cabinet, and electronic files were password‐protected and accessible only to the research team. All findings were reported in aggregate form to prevent the identification of individual participants.

### 2.5. Instruments

#### 2.5.1. Demographic and Clinical Characteristics

Demographic and clinical characteristics were collected using a researcher‐developed questionnaire based on relevant literature. Demographic variables included age, sex and education level. Clinical variables included diabetes duration, diabetes‐related complications and HbA1c.

#### 2.5.2. Charlson Comorbidity Index (CCI)

The CCI was used to assess comorbidity burden. The CCI is a diagnosis‐based weighted clinical index originally developed to classify comorbid conditions and estimate prognostic risk in longitudinal studies [20]. It is commonly used in clinical research to quantify comorbidity burden, with higher scores indicating a greater burden of comorbid disease [21]. In this study, comorbid conditions were identified from medical record data, and the total CCI score was calculated according to the standard scoring method.

#### 2.5.3. SARC‐F

Sarcopenia risk was assessed using the SARC‐F. The SARC‐F consists of five items evaluating strength, walking assistance, rising from a chair, climbing stairs and falls. Scores range from 0 to 10, with a score of ≥ 4 indicating a high risk of sarcopenia. As a screening instrument, the SARC‐F is intended to identify individuals at increased risk of sarcopenia and is not designed to establish a clinical diagnosis [22]. Previous studies have demonstrated acceptable reliability and construct validity of the SARC‐F in older adults and clinical populations [22, 23]. The Cronbach’s *α* value of the SARC‐F was 0.81 in the present study.

#### 2.5.4. Diabetes Distress Scale (DDS)

The DDS was used to assess diabetes‐related stress and emotional distress experienced during the previous month. The scale consists of 17 items and includes four subscales: Emotional Burden, Physician‐Related Distress, Regimen‐Related Distress and Interpersonal Diabetes‐Related Distress. Each item is rated on a 6‐point Likert‐type scale ranging from 1 (*no distress*) to 6 (*serious distress*). In this study, the overall DDS score was calculated by summing the scores of the 17 items and dividing by 17 to obtain the mean score, with higher scores indicating greater diabetes distress. According to the original scoring criteria, a mean score of < 2.0 indicates little or no distress, 2.0–2.9 indicates moderate distress and ≥ 3.0 indicates high distress [3]. The DDS has demonstrated good reliability and construct validity, with a Cronbach’s *α* of 0.87 for the overall scale [3, 10]. The Chinese version of the scale has also shown good internal consistency, with a Cronbach’s *α* of 0.88 [24]. In the present study, the Cronbach’s *α* of the overall scale was 0.90.

#### 2.5.5. Geriatric Depression Scale

The 15‐item Geriatric Depression Scale (GDS‐15) was used to assess depressive symptoms in older adults. Total scores range from 0 to 15, with higher scores indicating more severe depressive symptoms. Although a score of 5 or higher is commonly used as the cutoff point for depressive symptoms, the present study primarily used the total GDS score as a continuous variable in the inferential analyses. The GDS–15 has demonstrated good reliability and validity, with a Cronbach’s *α* of 0.84 and test–retest reliability of 0.84. The sensitivity and specificity for screening depressive symptoms in older adults are 95.5% and 84.2%, respectively [25, 26]. The Chinese version has been validated in older adults in Taiwan, with a Cronbach’s *α* of 0.91 and an area under the receiver operating characteristic curve of 0.83 [27]. In the present study, the Cronbach’s *α* of the scale was 0.89.

#### 2.5.6. Handgrip Strength Test

Handgrip strength was measured using a digital handgrip dynamometer (Camry, model EH101) with a qualified health department mark to assess muscle strength. According to the recommendations of the AWGS, participants stood with their arms hanging naturally and their elbows fully extended. Measurements were taken three times, with a 30‐s interval between measurements, and the maximum value was used for analysis [13, 28]. According to the AWGS sarcopenia criteria, handgrip strength below 28 kg in men and below 18 kg in women indicates low muscle strength [13].

### 2.6. Data Analysis

Data were analysed using SPSS Version 26.0. Descriptive statistics, including frequencies, percentages, means and standard deviations, were used to summarise participant characteristics and study variables. The prevalence of depressive symptoms was described using the conventional GDS–15 cutoff score of ≥ 5. For inferential analyses, the GDS total score was treated as a continuous dependent variable. Independent‐samples *t-*tests were used to examine differences in depressive symptom scores across categorical variables, and Pearson’s correlation coefficients were used to assess associations between continuous variables and depressive symptom scores. Variables that were significantly associated with depressive symptom scores in the univariate analyses were then entered into a stepwise multiple linear regression model to identify factors associated with depressive symptoms in older adults with T2DM. Multicollinearity among variables retained in the final regression model was assessed using variance inflation factors (VIFs). Missing data were handled using the listwise deletion method.

## 3. Results

### 3.1. Demographic and Clinical Characteristics

A total of 180 older adults with T2DM were recruited, of whom 10 withdrew for personal reasons, resulting in a final sample of 170 participants and a response rate of 94.4%. The mean age was 73.2 years (SD = 5.9), and 61.2% were male. Most participants (67.1%) had completed secondary education or below. The mean duration of diabetes was 9.9 years (SD = 5.1), and 73.5% had diabetes‐related complications. The mean number of comorbidities per participant was 1.6 (SD = 0.8). Additionally, 30.6% had an HbA1c level ≥ 7%, 18.2% were classified as high risk for sarcopenia, and 51.8% had low handgrip strength (Table 1).

**TABLE 1:** Demographic and clinical characteristics (*N* = 170).

Variable	*n* (%)	*M* (SD)
Age (year)		73.2 (5.9)
Sex		
Female	66 (38.8)	
Male	104 (61.2)	
Education level		
Secondary school or below	114 (67.1)	
High school or above	56 (32.9)	
Duration of diabetes (years)		9.9 (5.1)
Comorbidities (items)^∗^		1.6 (0.8)
Diabetics complications^∗∗^		
No	45 (26.5)	
Yes	125 (73.5)	
HbA1c (%)^∗∗∗^		
< 7	118 (69.4)	
≥ 7	52 (30.6)	
Risk of sarcopenia (SARC‐F)^†^		
Low (< 4)	139 (81.8)	
High (≥ 4)	31 (18.2)	
Handgrip strength (HSG)^‡^		
Normal	82 (48.2)	
Low	88 (51.8)	

^∗^Charlson comorbidity index item.

^∗∗^Diabetes complications, including diabetic nephropathy, arteriosclerosis, retinopathy, neuropathy and DM foot.

^∗∗∗^HbA1c ≥ 7 means poor glycaemic control.

^†^A SARC‐F questionnaire score of ≥ 4 points is a high risk of sarcopenia.

^‡^When the HSG of men is < 28 kg and that of women is < 18 kg, it indicates low handgrip strength.

### 3.2. Diabetes Distress and Depressive Symptoms

This study showed that the diabetes distress mean score in older adults with Type 2 diabetes was 2.76 points (SD = 1.3), and 7.1% of them had moderate distress. Interpersonal diabetes–related distress (20.6%) was the most common, followed by emotional burden (15.3%). In addition, 27 participants (15.9%) had depressive symptoms (Table 2).

**TABLE 2:** Diabetes distress and depressive symptoms (*N* = 170).

Variable	*n* (%)
DDS mean score (SD)	2.76 (1.3)
Diabetes distress level (DDS)^∗^	
No or mild	158 (92.9)
Moderate	12 (7.1)
High	0 (0)
Diabetes distress type	
Emotional burden	
No or mild	144 (84.7)
Moderate	22 (12.9)
High	4 (2.4)
Regimen‐related distress	
No or mild	154 (90.6)
Moderate	15 (8.8)
High	1 (0.6)
Physician‐related distress	
No or mild	167 (98.2)
Moderate	3 (1.8)
High	0 (0)
Interpersonal diabetes–related distress	
No or mild	135 (79.4)
Moderate	28 (16.5)
High	7 (4.1)
Depressive symptoms (GDS)^∗∗^	
Without	143 (84.1)
With	27 (15.9)

^∗^DDS, Diabetes Distress Scale; the intensity levels are mild or no distress (< 2.0), moderate distress (2.0–2.9) and high distress (≥ 3.0).

^∗∗^GDS, The Geriatric Depression Scale; GDS scores < 5 are considered without depression.

### 3.3. Univariate Analysis of Factors Associated With Depressive Symptom Scores

As shown in Table 3, depressive symptom scores were positively correlated with comorbidity (*r* = 0.28, *p* < 0.001) and diabetes distress mean score (*r* = 0.76, *p* < 0.001). Significant group differences in depressive symptom scores were also observed for the HbA1c group, sarcopenia risk and handgrip strength. Participants with HbA1c ≥ 7% had higher depressive symptom scores than those with HbA1c < 7% (2.58 ± 3.45 vs. 1.47 ± 2.69, *t* = −2.05, *p* = 0.044). Those at high risk of sarcopenia had higher depressive symptom scores than those without sarcopenia risk (4.23 ± 3.72 vs. 1.27 ± 2.50, *t* = −4.21, *p* < 0.001), and participants with low handgrip strength had higher depressive symptom scores than those with normal handgrip strength (2.30 ± 3.25 vs. 1.29 ± 2.57, *t* = −2.24, *p* = 0.027). No significant associations or group differences were found for age, duration of diabetes, gender, or diabetes complications (Table 3).

**TABLE 3:** Univariate analysis of factors associated with depressive symptom scores (*N* = 170).

Variable	Mean ± SD	*r*/*t*	*p*
Age		0.14	0.079
Duration of diabetes		0.05	0.532
Comorbidity (item)		0.28	< 0.001
DDS mean score		0.76	< 0.001
Gender		1.13	0.259
Female	2.14 ± 3.17		
Male	1.61 ± 2.84		
Diabetes complications		−1.47	0.145
No	1.33 ± 2.28		
Yes	1.98 ± 3.18		
HbA1c group		−2.05	0.044
< 7%	1.47 ± 2.69		
≥ 7%	2.58 ± 3.45		
High risk of sarcopenia		−4.21	< 0.001
No (0–3)	1.27 ± 2.50		
Yes (≥ 4)	4.23 ± 3.72		
Handgrip strength		−2.24	0.027
Normal	1.29 ± 2.57		
Low	2.30 ± 3.25		

*Note:* The dependent variable was the GDS‐15 total score. Continuous variables were examined using Pearson correlation coefficients (*r*), and dichotomous categorical variables were examined using independent‐samples *t*‐tests (*t*).

Abbreviations: DDS, Diabetes Distress Scale; SARC‐F, Sarcopenia Functional Questionnaire.

### 3.4. Factors Associated With Depressive Symptom Scores

A stepwise multiple linear regression analysis identified diabetes distress mean score, high risk of sarcopenia, low handgrip strength and comorbidity burden as significant factors associated with depressive symptom scores among older adults with T2DM. The final model was statistically significant (*F* = 94.14, *p* < 0.001) and explained 69.5% of the variance in depressive symptom scores, with an adjusted *R* ^2^ of 0.688. Among the retained variables, the diabetes distress mean score showed the strongest association (*β* = 0.71, *p* < 0.001), followed by high risk of sarcopenia (*β* = 0.28, *p* < 0.001), low handgrip strength (*β* = 0.12, *p* = 0.01) and comorbidity (*β* = 0.10, *p* = 0.03). Multicollinearity diagnostics showed that the VIF values of the variables retained in the final model ranged from 1.04 to 1.08, indicating no serious multicollinearity (Table 4).

**TABLE 4:** Stepwise multiple linear regression analysis for depressive symptoms among older adults with T2DM (*N* = 170).

Variable	*B*	SE	*β*	*t*	*p*	95% CI	VIF
Constant	−7.22	0.52	—	−13.91	< 0.001	−8.25, −6.20	
DDS mean score	5.94	0.37	0.71	15.87	< 0.001	5.20, 6.68	1.08
Risk of sarcopenia (high)	2.18	0.34	0.28	6.45	< 0.001	1.51, 2.85	1.05
Low handgrip strength	0.71	0.26	0.12	2.73	0.01	0.20, 1.22	1.04
Comorbidity (item)	0.31	0.14	0.10	2.14	0.03	0.02, 0.59	1.07

*Note:* The dependent variable was the GDS‐15 total score. *B* = unstandardised regression coefficient, *β* = standardised regression coefficient. The VIF values ranged from 1.04 to 1.08, indicating no serious multicollinearity. Model statistics: *F* = 94.14, *p* < 0.001, adjusted *R* ^2^ = 0.69.

Abbreviations: CI, confidence interval; DDS, Diabetes Distress Scale; SE, standard error; VIF, variance inflation factor.

## 4. Discussion

This study examined factors associated with depressive symptom severity among older adults with T2DM. The final stepwise multiple linear regression model identified diabetes distress, high sarcopenia risk, low handgrip strength, and comorbidity burden as significant associated factors. Among these, diabetes distress showed the strongest association. These findings suggest that depressive symptom severity in older adults with T2DM is related to psychological distress, functional vulnerability, reduced muscle strength and overall disease burden. Therefore, assessment of depressive symptoms in this population should consider psychological, functional and clinical factors simultaneously.

Participants with higher diabetes distress scores reported more severe depressive symptoms. This finding is consistent with previous studies showing that diabetes distress is closely associated with depressive symptoms among adults with T2DM [5, 9, 29]. Diabetes distress reflects the emotional burden related to long‐term diabetes self‐management, including concerns about glycaemic control, treatment demands, interpersonal support and communication with healthcare professionals. For older adults with T2DM, these ongoing self‐care demands may coexist with age‐related health problems, functional limitations, dependence on others and greater psychological vulnerability. Therefore, diabetes distress should not be viewed merely as a transient emotional response but also as an important psychological factor that may coexist with more severe depressive symptoms in older adults with T2DM.

Notably, interpersonal distress was the most common type of diabetes distress in this study, followed by emotional burden. This finding differs from previous studies in which emotional burden was reported as the most common type of diabetes distress [30, 31]. One possible explanation is that older adults with diabetes may have gradually adapted to the biomedical aspects of the disease, especially after living with diabetes for several years, but may become increasingly dependent on family members, healthcare professionals, or social networks for daily disease management. Insufficient understanding, inadequate support, communication difficulties, or interpersonal tension may therefore become more prominent sources of diabetes‐related distress. This finding suggests that interpersonal support should be considered an important component of diabetes care for older adults. In clinical practice, healthcare professionals should not only assess individual emotional burden but also explore patients’ perceived support from family members, caregivers, and healthcare providers.

High sarcopenia risk was significantly associated with depressive symptom severity. Older adults with T2DM who were at high risk of sarcopenia reported higher depressive symptom scores than those with lower sarcopenia risk. This finding is consistent with previous studies suggesting that sarcopenia and sarcopenia‐related functional decline are associated with poorer mental health outcomes, including depressive symptoms [8, 15, 17]. Sarcopenia risk may reflect broader functional limitations, such as difficulty walking, rising from a chair, or climbing stairs and a history of falls. These limitations may be accompanied by restricted daily activities, reduced social participation, greater dependence on others and poorer perceived health. Among older adults with T2DM, sarcopenia risk may therefore represent an important functional vulnerability associated with depressive symptom severity.

Low handgrip strength was also associated with depressive symptom severity. This finding is consistent with previous evidence showing that reduced muscle strength is related to depressive symptoms among older adults with T2DM [18]. Handgrip strength is a practical indicator of muscle strength and is included in sarcopenia assessment recommendations. According to the AWGS, sarcopenia assessment involves muscle strength, muscle mass and physical performance, and handgrip strength is commonly used as a feasible measure of muscle strength in clinical and research settings [13]. Although sarcopenia risk and handgrip strength are conceptually related, they may reflect different aspects of physical function. Sarcopenia risk, as measured by the SARC‐F, captures broader self‐reported functional limitations, whereas handgrip strength reflects objectively measured muscle strength. Their simultaneous retention in the final model suggests that both perceived functional limitations and measured muscle strength provide complementary information regarding depressive symptom severity.

Comorbidity burden was also associated with depressive symptom severity. This finding is partly consistent with Fung et al. [7] and Dziedzic et al. [6], who identified comorbidity as an independent factor associated with depressive symptoms among older adults with T2DM. A greater comorbidity burden may reflect more complex health problems, greater symptom burden, higher treatment demands, polypharmacy, increased healthcare utilisation and greater functional limitations. These conditions may coexist with psychological distress and higher depressive symptom severity. Therefore, depressive symptoms among older adults with T2DM should not be interpreted solely in relation to diabetes‐specific indicators but should also be understood within the broader context of multimorbidity and overall health burden.

In contrast, age, sex, duration of diabetes, and HbA1c were not retained in the final model. Previous evidence regarding these factors remains inconsistent. Some studies have reported no significant associations between selected demographic or disease‐related variables and depressive symptoms among older adults with T2DM [5, 7], whereas other studies have suggested that female sex and longer diabetes duration may be associated with depressive symptoms in certain populations [6, 8, 32]. Similarly, evidence regarding the association between glycaemic control and depressive symptoms remains inconclusive. Some studies have reported that poor glycaemic control is associated with depressive symptoms [7, 25], whereas others found no significant association between HbA1c and depressive symptoms [6, 8, 33]. In the present study, participants with HbA1c ≥ 7% had significantly higher depressive symptom scores than those with HbA1c < 7% in the univariate analysis; however, HbA1c was not retained in the final stepwise regression model. Previous evidence suggests that glycaemic control, diabetes distress and depressive symptoms are interrelated, with diabetes distress potentially mediating the relationship between depressive symptoms and glycaemic control [34, 35], suggesting that glycaemic control alone may not fully explain depressive symptom severity.

Taken together, these findings highlight the need to consider psychological and functional factors in addition to biomedical indicators when assessing depressive symptoms and related care needs in this population. Although glycaemic control remains clinically important, clinical assessment should also incorporate diabetes distress, interpersonal support, sarcopenia risk, muscle strength and multimorbidity. Such an integrated approach may help healthcare professionals to identify older adults who report more severe depressive symptoms and may require further psychological or functional assessment.

### 4.1. Clinical Implications

For nursing practice, the findings suggest that care for older adults with T2DM should integrate psychological assessment, diabetes self‐management support, interpersonal support evaluation and physical function screening. Nursing care may incorporate emotional support, patient‐centred diabetes education, family involvement, referral for mental health evaluation when needed and strategies to support muscle strength and functional independence.

## 5. Conclusion

In conclusion, depressive symptom severity among older adults with T2DM was associated with diabetes distress, high sarcopenia risk, low handgrip strength, and greater comorbidity burden, with diabetes distress showing the strongest association. These findings support the need for integrated diabetes care that includes assessment of depressive symptoms, diabetes distress, sarcopenia risk, muscle strength and comorbidity burden. Early identification of these psychological, functional, and clinical vulnerabilities may help nurses and multidisciplinary teams provide timely supportive care for older adults with T2DM.

## 6. Limitations and Recommendations

This study has several limitations. First, the cross‐sectional design limits causal inference, and convenience sampling may reduce the generalisability of the findings. Future studies should use probability sampling and longitudinal designs to clarify temporal relationships among diabetes distress, sarcopenia risk, muscle strength, comorbidity burden and depressive symptoms. Second, this study assessed sarcopenia risk rather than confirmed sarcopenia diagnosis because objective measures of appendicular skeletal muscle mass, such as dual‐energy X‐ray absorptiometry or bioelectrical impedance analysis, were not used. Therefore, the definition of high sarcopenia risk in this study may differ from internationally recognised diagnostic criteria for sarcopenia, which may affect the interpretation of the findings and comparisons with studies using diagnostic criteria. Future studies should incorporate objective assessments of muscle mass and physical performance. Third, although both high sarcopenia risk and abnormal handgrip strength were retained in the final model, these variables are conceptually related; future research should further examine their distinct and combined contributions. Fourth, depressive symptoms were assessed using a screening tool rather than a formal clinical diagnosis, and the findings should therefore be interpreted as associations with depressive symptom severity rather than clinically diagnosed depression. Fifth, this study did not examine psychological resilience [29] or other psychosocial factors as potential mediators or moderators. Last, because stepwise regression is sensitive to sample characteristics and variable selection procedures, the final model should be interpreted as exploratory and validated in independent samples.

## Funding

No funding was received for this manuscript.

## Conflicts of Interest

The authors declare no conflicts of interest.

## Supporting Information

Additional supporting information can be found online in the Supporting Information section.

## Supporting information


**Supporting Information** STROBE statement: a checklist of items that should be included in reports of cross‐sectional studies.

## Data Availability

The datasets generated or analysed during this study are available from the corresponding author on reasonable request.
